# A case of multiple myeloma presenting as a distal renal tubular acidosis with extensive bilateral nephrolithiasis

**DOI:** 10.1186/s12878-016-0047-7

**Published:** 2016-03-17

**Authors:** Chathuranga Lakmal Fonseka, Sampath Rukshani Galappaththi, Jeewandarage Dhanushka Karunarathna, Dayakshi Dushyantha Kumarihami Abeyaratne, Nirmali Tissera

**Affiliations:** General Medicine, National Hospital, Colombo, Sri Lanka

**Keywords:** Multiple Myeloma, Distal renal tubular acidosis, Nephrolithiasis

## Abstract

**Background:**

At the time of diagnosis, Multiple Myeloma is commonly associated with renal impairment. Renal tubular acidosis without overt renal insufficiency is an uncommon disease presentation of myeloma. Among tubular acidosis types, isolated renal tubular acidosis is a very unusual presentation of multiple myeloma.

**Case presentation:**

We present a 55 years old female who presented with lower limb weakness due to persistent hypokalaemia caused by distal renal tubular acidosis. On further investigation of her anaemia with high erythrocyte sedimentation rate, we diagnosed IgG myeloma.

**Conclusion:**

Isolated distal renal tubular acidosis is a rare presentation of multiple myeloma. In the absence of hypercalciuria and demonstrable light chain excretion in urine, we assumed that the distal renal tubular acidosis could have been caused by monoclonal hypergammaglobulinaemic state of multiple myeloma.

## Background

Renal Tubular Acidosis (RTA) is an infrequent finding associated with Multiple Myeloma. Out of the types of renal tubular acidosis, proximal RTA is well known to be associated with myeloma. It occurs due to the toxic effects of excreted light chains on proximal renal tubular cells leading to tubular dysfunction without overt renal insufficiency. However, isolated distal tubular acidosis due to multiple myeloma is very unusual and is only reported in few case studies. The underlying pathogenesis of distal RTA secondary to multiple myeloma is not well described in literature.

We report a case of Multiple Myeloma leading to distal tubular acidosis and bilateral nephrolithiasis without renal impairment. In our case, there was no demonstrable light chains detected in urine and there was no evidence of hypercalciuria to cause distal tubular involvement. Therefore, in the absence of the above, our patient’s distal tubulopathy could be a result of monoclonal hypergammaglobulinaemic state caused by multiple myeloma.

## Case presentation

We report a 55 year old female, who was diagnosed to have chronic autoimmune hypothyroidism for past 5 years on thyroxine, presented with insidious onset of constitutional symptoms for 6 months duration. Her thyroid function tests showed satisfactory control. Despite satisfactory control of thyroid function tests, she experienced a gradual but profound loss of weight of thirty six (36 kg) kilograms with loss of appetite for the same duration. She did not have any past or contact history of tuberculosis or a past history of malignant disease. She is not on any medication too. Over the last few months, she was complaining of a worsening generalized malaise, which affected her activities of daily living. There was no muscle pain but complained that her walking has become clumsy and it was difficult for her to stand from the seated position. On examination, she was cachectic with BMI of 19 kg/m^2^ and was found to be pale, without icterus or palpable lymph nodes or organomegaly. Neurological examination revealed reduced muscle power in lower limbs with diminished reflexes without any sensory deficit. Her fundoscopic examination was normal.

Her initial investigations revealed normocytic normochromic anaemia (8.9 g/dl) with rouleaux formation. She had elevated ESR of 120 mm/1^st^ hour and hypokalemia (2.9 mmol/l). Liver function tests showed reversed albumin to globulin ratio (Albumin – 29 g/l, Globulin – 50 g/l). On further investigation, serum protein electrophoresis revealed a monoclonal band in the gamma region. However, her skeletal survey and urinary Bence Jones protein were negative. In bone marrow aspiration, there were 15 % clonal plasma cells and immunofixation revealed IgG myeloma.

**Fig. 1:**
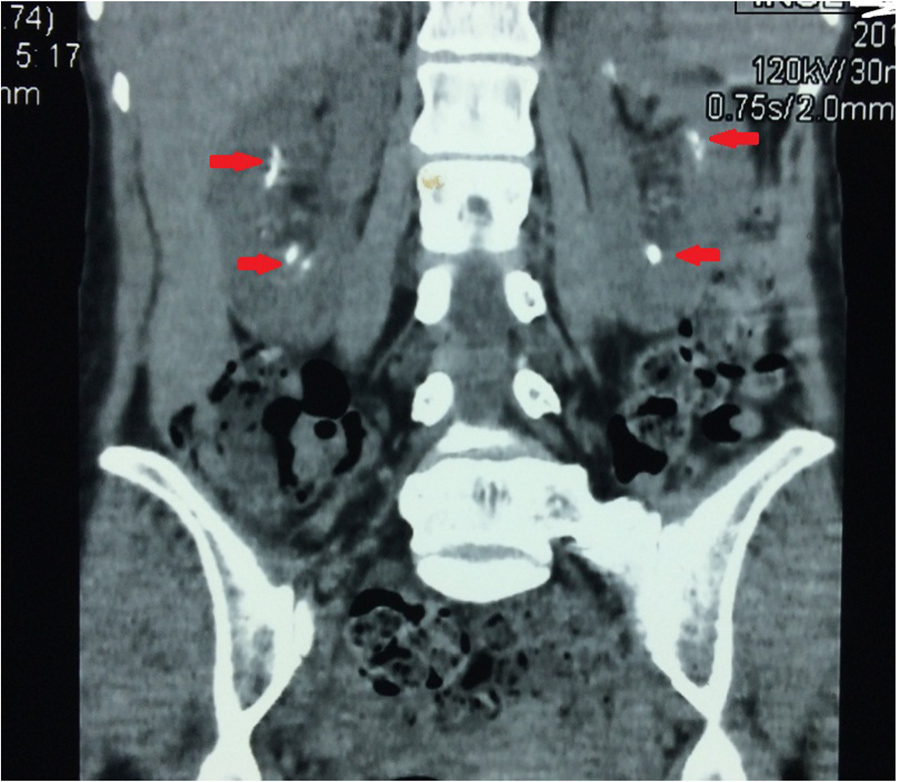
Non-contrast CT kidneys showing bilateral nephrolithiasis

After confirming the low potassium, we detected her urinary excretion of potassium to be increased. Furthermore, her arterial blood gas showed a marked hyperchloraemic metabolic acidosis (pH 7.2, HCO_3_- 8.2 mmol/l) with normal anion gap (15 mmol/l). Her urine pH was 7.5 and was high despite systemic acidosis and the fractionated bicarbonate excretion was 3 %. Her non-contrast CT of kidneys revealed bilateral non-obstructing renal calculi (Fig. 1).

Her autoimmune markers namely ANA, Rheumatoid factor, Complement levels were all normal. Her serum ionized calcium level (1.1 mmol/l) and urinary excretion of oxalate, phosphate, uric acid, calcium and citrate levels were within normal range. Contrast CT scan of chest and abdomen did not show evidence of sarcoidosis or malignancy. The summary of the investigations is depicted in Table 1.

**Table 1:** Summary of investigations

Urinalysis		Arterial blood gas	
Urine pH	7.5	pH	7.2
Protein	Nil	Bicarbonate	8.2 mmol/l
Glucose	Nil	pCO2	20 mmHg
Pus cells	Few	Base excess	−17
Red cells	1–2	Anion gap	15 mmol/l
Casts	Nil	Fractionated bicarbonate excretion	3 %
Crystals	Nil	ANA	Negative
Urinary anion gap	Negative	Complement levels (C_3_, C_4_)	Normal
Urinary Sodium	32 mmol/l	Rheumatoid factor	Negative
Urinary Pottasium	45 mmol/l	24 h urinary excretion	
Blood Biochemistry		Oxalate	0.21 mmol/d (0.04–0.32)
Na	145 mmol/l	Phosphate	25.6 mmol/d (12.9–42)
K	2.9 mmol/l	Uric acid	476 mg/d (250–750)
Cl	115 mmol/l	Calcium	5.1 mmol/d (2.5–7.5)
Glucose	105 mg/dl	Citrate	720 mg/day (>600)
Total Protein	88 g/l	Serum protein electrophoresis	
Albumin	29 g/l	Monoclanal band in gamma region	
Globulin	59 g/l	Paraproteinaemia	36.7 g/l
Creatinine	82 μmol/l	Immunofixation	IgG Kappa Myeloma
Ionized calcium	1.1 mmol/l	Bone marrow aspiration	
ALP	147 U/l	15 % bone marrow clonal plasma cells	
Chest radiograph	Normal		
Contrast CT chest and abdomen		Urine protein electrophoresis & Immunofixation	
Bilateral nephrolithiasis		Absent light chains in urine	
Mantoux	Negative		

## Discussion

Renal involvement is a common finding in the diagnosis of multiple myeloma, where nearly 30–40 % of patients were found to have raised serum creatinine levels [1]. Myeloma can damage the kidney at multiple levels: namely glomerular, tubular and interstitial. Two major causes of renal insufficiency in patients with myeloma are light chain cast nephropathy and hypercalcemia. Apart from that, renal insufficiency occurs due to light chain (AL) amyloidosis, light chain deposition disease, interstitial nephritis or could be drug-induced. However, in rare occasions, monoclonal heavy chain deposition and non-monoclonal protein related renal injury may also occur [2].

Out of the various renal manifestations of myeloma, renal tubular acidosis is an uncommon entity. Although adult proximal tubular dysfunction is well described as an association of myeloma; distal renal tubular acidosis (dRTA) in the background of multiple myeloma is extremely rare. It is described only in few reports in literature. Proximal tubules are affected during the process where the filtered light chains get reabsorbed and catalyzed within the epithelial cells. These epithelial cells get damaged with the activation of lysosomal enzymes and from direct toxicity of light chains [3–5]. Distal tubules are thought to be affected due to either hypercalciuria or hypergammaglobulinaemia (usually polyclonal) [6, 7].

Among the case series of RTA with multiple myeloma, DeFronzo et al [8] described in his series of 14 patients with urinary acidification defects: two had distal tubular involvement. Another Czech case series showed that eight out of 21 patients developed distal renal tubular defects [9]. Out of the three case reports which described the association of dRTA and myeloma, first case report, described a patient having early myeloma with IgG lambda chains who developed distal RTA [5] and second, a patient with Sjogrens’ syndrome subsequently developed monoclonal gammopathy of unknown significance (MGUS) with dRTA [10]. Third report described a patient with IgA kappa multiple myeloma with both proximal and distal renal tubular involvement, probably due to the overflow of light chains from proximal to the distal renal tubules and deposition in the renal tubular epithelial cells [11]. But the pathogenesis of dRTA was unclear in the above case reports as they neither had hypercalciuria nor polyclonal hypergammaglobulinaemia. But they had monoclonal gammaglobulinaemia which may have caused dRTA in the same mechanism in which polyclonal hypergammaglobulinaemia caused dRTA.

In our patient, the diagnosis of dRTA was made by the presence of hypokalemia, hyperchloremic metabolic acidosis with high urinary pH above 5.5. Distal tubular involvement was confirmed by the presence of low fractionated bicarbonate excretion and its association with bilateral nephrolithiasis or nephrocalcinosis [11]. There was no proximal tubular involvement in our patient. It is mandatory to find a secondary cause for dRTA, since concomitant treatment of RTA and the underlying cause is important for a better prognosis [12]. Therefore, we searched for a cause for the dRTA in our patient, in the background of anaemia and high ESR. Autoimmune panel, ionized calcium levels, CT scan of chest and abdomen, transaminases and other screening for granulomatous diseases were all negative. But, investigation of bone marrow and serum protein electrophoresis led to the diagnosis of multiple myeloma. Immunofixation showed a monoclonal peak (36.7 g/l) which could be the cause for dRTA. Unfortunately, free light chain assay was not readily available, hence urine immunofixation was performed and it showed absent overt light chain excretion.

Pathogenesis of isolated dRTA due to myeloma is not well described earlier. It is highly unlikely that light chain deposition would cause to dRTA, as it’s known to cause proximal RTA. Previous studies have demonstrated an association between hypergammaglobulinemia and distal RTA [8, 13]. But the hypergammaglobulinamia, which is associated with dRTA, is usually polyclonal [14]. Feringa et al have described a case of Waldenstrom’s Macroglobulinaemia, in which IgM monoclonal hyperglobulinaemia associated with dRTA [14]. Therefore, in the absence of hypercalciuria in our patient, multiple myeloma induced monoclonal hypergammaglobulinaemia would have led to dRTA. Unfortunately, renal biopsy has not been performed on her, so that the exact pathogenesis was difficult to ascertain.

Distal RTA can lead to bilateral nephrolithiasis due to hypercalciuria, hyperphosphaturia, hypocitraturia, and low urine pH. In the absence of hypercalciuria, hyperphosphaturia, hypocitraturia, we assumed that urine pH has led to the extensive stone formation in our patient [15].

Having arrived at the diagnosis of dRTA, we started her on oral bicarbonate and oral potassium chloride. Her lower limb weakness showed a marked improvement with the normalization of potassium. Subsequently, with correction of acidosis we could tail off potassium supplementation. After obtaining Oncology opinion, standard chemotherapy was initiated as the treatment for myeloma and at 3 months of completion of chemotherapy, her bicarbonate requirement and the inflammatory markers were noted to be reduced and the monoclonal band peak also reduced to 7.2 g/l. In our attempt of managing this patient, we assume that the treatment of myeloma can improve the monoclonal gammopathy as well as the RTA.

## Conclusion

Our case illustrates that Multiple Myeloma can rarely present as an isolated distal renal tubular acidosis without overt renal insufficiency. Our investigations demonstrated that the monoclonal hypergammaglobulinaemia secondary to myeloma would have been the probable cause for the dRTA in this patient. As the evidence of a renal biopsy was not available, exact pathogenesis cannot be explained, with certainty. With our experience, it was evident that the treatment of myeloma could improve both myeloma per se and the dRTA.

## Consent

Written informed consent was obtained from the patient for publication of this Case report and images. Approval was granted from the Ethical Review Committee of National Hospital of Sri Lanka for publishing the case report.
